# Segmental Abnormalities of White Matter Microstructure in End-Stage Renal Disease Patients: An Automated Fiber Quantification Tractography Study

**DOI:** 10.3389/fnins.2021.765677

**Published:** 2021-12-06

**Authors:** Yuhan Jiang, Yangyingqiu Liu, Bingbing Gao, Yiwei Che, Liangjie Lin, Jian Jiang, Peipei Chang, Qingwei Song, Nan Wang, Weiwei Wang, Yanwei Miao

**Affiliations:** ^1^Department of Radiology, The First Affiliated Hospital of Dalian Medical University, Dalian, China; ^2^Department of Radiology, The Third People’s Hospital of Dalian, Dalian, China; ^3^Philips Healthcare China, Beijing, China; ^4^Department of Nephrology, The First Affiliated Hospital of Dalian Medical University, Dalian, China

**Keywords:** white matter microstructure, end-stage renal disease, diffusion tensor imaging, automated fiber quantification, serum urea, uric acid

## Abstract

**Background and Purpose:** End-stage renal disease (ESRD) results in extensive white matter abnormalities, but the specific damage segment cannot be identified. This study aimed to determine the segmental abnormalities of white matter microstructure in ESRD and its relationship with cognitive and renal function indicators.

**Methods:** Eighteen ESRD patients and 19 healthy controls (HCs) were prospectively recruited. All participants underwent DTI and clinical assessments. Automatic fiber quantification (AFQ) was applied to generate bundle profiles along 16 main white matter tracts. We compared the DTI parameters between groups. Besides, we used partial correlation and multiple linear regression analyses to explore the associations between white matter integrity and cognitive performance as well as renal function indicators.

**Results:** In the global tract level, compared to HCs, ESRD patients had greater MD, AD, and RD values and lower FA value in several fibers (*P* < 0.05, FDR correction). In the point-wise level, extensive damage existed in specific locations of different fiber tracts, particularly in the left hemisphere (*P* < 0.05, FDR correction). Among these tracts, the mean AD values of the left cingulum cingulate correlated negatively with MoCA score. Urea and UA level were independent predictors of the AD value of superior component of the left corticospinal. Besides, urea level was the independent predictors of mean MD value of left anterior thalamic radiation (ATR).

**Conclusion:** White matter fiber tract damage in ESRD patients may be characterized by abnormalities in its specific location, especially in the left hemisphere. Aberrational specific located fibers were related to cognitive impairment and renal dysfunction.

## Introduction

Chronic kidney disease (CKD) is a condition characterized by a gradual loss of kidney function. Estimated glomerular filtration rate (eGFR) is an objective indicator for evaluating glomerular filtration capacity and is usually used to reflect renal function. End-stage renal disease (ESRD) is the final stage of chronic kidney disease (CKD), which is defined as an eGFR of less than 15 ml/min/1.73 m^2^. ESRD patients require renal replacement therapy via maintenance dialysis or kidney transplantation to maintain life (National Kidney Foundation, 2002; Romagnani et al., 2017). CKD induces a series of pathophysiological processes which in turn affect the structural and functional integrity of the brain, which is more pronounced in ESRD patients (Lu et al., 2015). In addition, ESRD patients are often accompanied by cognitive decline. A study has confirmed that ESRD is an independent risk factor for cognitive impairment (O’Lone et al., 2016).

Neuroimaging research has greatly promoted our understanding of the ESRD-related neurological changes. Most of the previous quantitative analyses focused on the abnormal structure of gray or white matter (Zhang et al., 2013; Murea et al., 2015; Meurs et al., 2016). In recent years, more and more studies have confirmed that the integrity of white matter microstructure in ESRD patients has also changed using various analytical methods based on diffusion tensor imaging (DTI), including region of interest (ROI), voxel-based analysis (VBA), and tract-based spatial statistics (TBSS) (Chou et al., 2013, 2019; Kong et al., 2014; Chen et al., 2015; Yin et al., 2018; Liu et al., 2020). However, the location and range of decrease of white matter integrity varied differently across studies. For example, a ROI-based study found that compared with the control group, FA values in all regions were reduced (Hsieh et al., 2009). Results from VBA studies showed FA reduction within the middle cerebellar peduncle, bilateral sagittal stratum, and the genu and splenium of the corpus callosum (Chou et al., 2013). TBSS results have shown that lower FA values within the bilateral corona radiata (Kong et al., 2014; Yin et al., 2018), bilateral inferior fronto-occipital fasciculus (IFOF) (Eldehni et al., 2019), bilateral superior longitudinal fasciculus (SLF) (Zhang et al., 2015; Yin et al., 2018), left anterior thalamic radiation (ATR) (Zhang et al., 2015), the body of the corpus callosum (Drew et al., 2017), and bilateral inferior longitudinal fasciculus (ILF) (Eldehni et al., 2019). In addition, ROI-based analysis lacks consistent standard and largely dependent on the personal evaluation. Because of the differences in the shape of the white matter fiber bundles between subjects, VBA is not accurate enough at the individual level (Wassermann et al., 2011). Although TBSS can be more accurate than VBA, it still fails to provide specific properties for positioning the white matter integrity along each fiber bundle (Bach et al., 2014). Automatic fiber quantification (AFQ) is a new algorithm that can automatically identify the main fiber bundles, allowing researchers to quantify diffusion indicators at anatomically equivalent locations along the fiber tracts (Yeatman et al., 2012). This tract-oriented automatic quantization approach provides more detailed diffusion parameters when mean measurement along each tract is not obvious.

To our best knowledge, this is the first study of applying the AFQ technique to explore potential regional white matter fiber alterations in ESRD patients. We hypothesize that abnormal white matter integrity existed in specific regions of tracts in ESRD patients. We also explored the relationships of DTI metrics with clinical and laboratory characteristics to further investigate the locations of brain structure changes that are associated with certain clinical manifestations.

## Materials and Methods

### Participants

This is a prospective study approved by the ethics committee of the first affiliated hospital of our Medical University, and written informed consent was obtained from each participant. The inclusion criteria for ESRD were: (a) confirmed ESRD diagnosis by nephrologist based on the kidney disease outcomes quality initiative (K/DOQI) classification; (b) maintenance hemodialysis (3–4 times per week) for at least 3 months; (c) right-handedness; and (d) age > 18 years. Exclusion criteria included: (a) diagnosis of psychiatric or neurological disorders (e.g., infarction), traumatic brain injury; (b) recipient of renal transplant or acute renal failure (ARF); and (c) contraindications for MRI examination (e.g., claustrophobia, pacemaker).

Between April 2019 and June 2021, 23 patents diagnosed with ESRD were prospectively enrolled. Patients with poor image quality (*n* = 3) and with claustrophobia (*n* = 2) were excluded. Therefore, 18 (10 men; 8 women; mean age, 57.28 years; age range, 33–74) ESRD patients were enrolled in the final analysis. Nineteen healthy controls (8 men; 11 women; mean age, 56.05; age range, 34–75; HCs, right-handedness) were recruited with similar gender, age, body mass index (BMI), and education level to the ESRD patients. The exclusion criteria for HCs were traumatic brain injury, mental, and neurological disorders.

### Magnetic Resonance Imaging Acquisition

Magnetic resonance imaging (MRI) scans were performed using a 3.0-T MRI scanner (Ingenia CX, Philips Healthcare, Best, the Netherlands) equipped with a 32-channel phased-array head coil. High-resolution, three-dimensional (3D), T1-weighted (T1W) images were obtained using a multi-shot turbo field echo (MS-TFE) sequence with the following scan parameters: echo time (TE) = 3.0 ms, repetition time (TR) = 6.6 ms, flip angle (FA) = 12°, slices = 188, field of view (FOV) = 256 × 256 mm^2^, matrix size = 256 × 256, and thickness = 1.0 mm. We obtained the DTI data using a single-shot echo planar imaging (SS-EPI) sequence (TE = 92 ms, TR = 6,000 ms, FA = 90°, voxel size = 2 × 2 × 2 mm^3^, FOV = 256 × 256 mm, matrix size = 128 × 128, 68 axial slices of 2 mm thickness to cover the whole brain without gap). Each DTI dataset included 64 non-collinear spatial directions at *b-*value = 1,000 s/mm^2^ and one baseline image at *b* = 0 s/mm^2^.

### Data Preprocessing

Diffusion-weighted images were preprocessed by the open-source Vistasoft package version 1.0.^1^ Eddy current-induced distortion correction, motion artifact correction, and skull stripping were all performed using the Functional MRI of the Brain (FMRIB) Software Library (FSL) version 5.0.9^2^ (Smith et al., 2004). A diffusion tensor model at each voxel was fitted using DTIFIT command of FSL to generate fractional anisotropy (FA), mean diffusivity (MD), eigenvalue (λ1, λ2, λ3) maps and a raw T2 image without diffusion weighting (S0). Then the axial (AD = λ1) and radial [RD = (λ2 + λ3)/2] diffusivity maps were calculated for each participant. For the 3D T1W scans, firstly, using “Brain Extraction Tool (BET),” a function of the FSL, to remove non-brain structures. Then, the T1W images were averaged and rotated to align with the anterior commissure–posterior commissure (AC-PC) plane using FSL. Finally, the script *dtiMakeDt6FromFSL* was used to obtain a *dt6* MATLAB format file by aligning the T1W image to the S0 image.

### Automated Fiber Quantification

**FIGURE 1:**
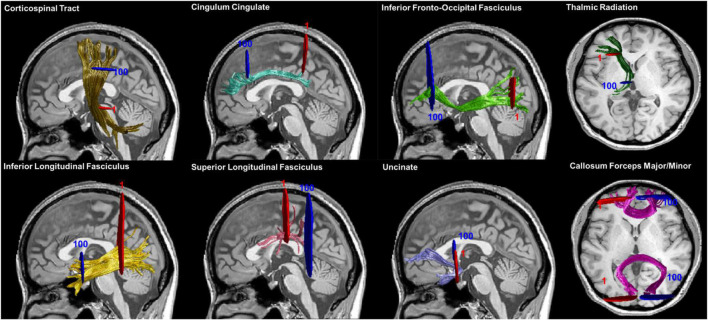
Fiber tracts identification results. Sixteen main fiber tracts were successfully identified by AFQ. Starting and ending waypoint ROIs are described in red and blue, respectively. Abbreviation: ROIs, regions of interest.

Twenty major fiber tracts of the whole brain were preliminarily identified and we quantified the diffusion metrics along the tracts by applying an open-source MATLAB version of AFQ (Yeatman et al., 2012). The identification procedure included the following procedures: (1) whole-brain deterministic fiber tractography using a streamline tracking algorithm with thresholds of FA > 0.2 and turning angle < 30°; (2) waypoint ROI-based tract segmentation (Wakana et al., 2007); (3) fiber tracts refinement based on the probabilistic fiber tract maps (Hua et al., 2008); (4) fiber tract cleaning using an iterative procedure to remove fibers more than four standard deviations (SDs) above the mean fiber length or far from the core of the fiber bundle (Yeatman et al., 2012); and (5) quantification of the DTI metrics of each participant at 100 equidistant nodes along each fiber tract. However, we failed to identify some fibers in large portion of participants due to the strict criterion for tract segmentation, including the bilateral arcuate fasciculus (AF) and bilateral cingulum hippocampus (CH). Therefore, four fiber tracts were excluded from the subsequent analysis. The fully identified 16 fiber tracts were showed in Figure 1.

### Neurocognitive Assessments and Clinical/Laboratory Tests

Among all participants, only 12 ESRD patients and 13 HCs completed cognitive assessment [Beijing revised version Montreal Cognitive Assessment (MoCA)] before MR data acquisition.

All ESRD patients underwent several biochemical tests, including serum urea (urea), uric acid (UA), creatinine (Cre), cystatin C (Cys C), homocysteine (HCY), low density lipoprotein (LDH), high density lipoprotein (HDL), and triglyceride (TG) levels before MR data acquisition. Participants in the HCs group did not undergo biochemical tests.

### Statistical Analyses

SPSS Statistics V22.0. was used to analyze the demographic characteristics (including age, gender, BMI, and years of education) and cognitive assessment (MoCA score) from the HC individuals and ESRD patients. We used two-sample *t*-tests or Mann–Whitney *U*-tests to compare normally or non-normally distributed variables, respectively. Chi-squared (χ^2^) tests or Fisher’s exact tests were performed in the comparisons of categorical data. The significant threshold was set at corrected *P* < 0.05.

First, we compared the group differences of WM tracts from the level of global tract; average DTI metrics (FA, MD, AD, and RD) were calculated by averaging these diffusion values of 100 nodes along each fiber tract and the two-sample *t*-test was performed to determine the differences.

Then, we applied the point-wise analyses based on the “Randomize” command in FSL, controlling gender, age, BMI, and years of education as covariates in the general linear model (GLM). A non-parametric permutation-based statistical analysis (5,000 permutations), with false discovery rate (FDR) procedure (*q* = 0.05) for multiple comparisons was utilized to the 1,600 points and only significant differences observed at ≥ 3 adjacent nodes were reported (Banfi et al., 2019).

Correlation analysis between diffusion properties and MoCA scores as well as laboratory tests was performed using partial Pearson correlation, with age, gender, BMI, and education as covariate. FDR correction was applied to reduce the false-positive errors. We then performed multiple linear regression analysis to find independent predictors for explaining the relationship between laboratory tests and DTI metrics. Statistical tests were two-tailed, and *P* < 0.05 was considered statistically significant.

## Results

### Demographic and Clinical Characteristics

The demographic and clinical characteristics of the ESRD patients and HCs are summarized in Table 1. Age, gender, BMI, and years of education between groups showed no statistical difference (*P* > 0.05). The incidence of hypertension in ESRD patients is higher (*P* = 0.033). Meanwhile, poorer performances on MoCA (*P* = 0.018) were found in ESRD group, especially in delayed recall (*P* = 0.005).

**TABLE 1:** Demographic and clinical characteristics.

	HC	ESRD	t/Z/χ^2^	*P*-value
Age, years	56.05 ± 11.65	57.28 ± 12.22	–0.312	0.757
Gender (male/female)	8/11	10/8	0.669	0.413
Education, years, M(IQR)	12 (12, 15)	13 (9.75, 15)	–0.094	0.925
BMI, kg/m^2^	24.25 ± 2.42	23.63 ± 2.78	0.716	0.479
Hypertension, *n* (%)	6 (31.58)	15 (83.33)	−	0.003*
Diabetes mellitus, *n* (%)	5 (27.78)	9 (50.00)	−	0.535
History of smoking, *n* (%)	6 (31.58)	4 (22.22)	−	0.714
History of drinking, *n* (%)	4 (21.05)	3 (16.67)	−	1.000
MoCA, *n*, M(IQR)	13, 26 (25, 28)	12, 23.5 (22, 24.25)	–2.894	0.004*
Visuospatial/executive, M(IQR)	5 (3, 5)	4 (3, 4)	–1.104	0.320
Naming, M(IQR)	3 (3, 3)	3 (2.75, 3)	–1.155	0.248
Attention, M(IQR)	6 (5, 6)	5 (4, 6)	–1.239	0.215
Language, M(IQR)	2 (1, 2)	2 (1, 2)	–0.237	0.812
Abstraction, M(IQR)	2 (2, 2)	2 (1.75, 2)	–0.311	0.756
Delayed recall, M(IQR)	4 (4, 5)	2.5 (2, 3)	–3.056	0.002*
Orientation, M(IQR)	6 (6, 6)	6 (5.75, 6)	–1.883	0.060

*HC, health controls; BMI, body mass index; ESRD, end stage renal disease; M, median; n, number; IQR, interquartile range.*

*Values are presented as mean ± standard deviation (SD), n (%), or M (IQR).*

*Represents the statistical difference between the two groups, P < 0.05.

### Group Difference in Global Tract Level and Point-Wise Level

#### Fractional Anisotropy

**FIGURE 2:**
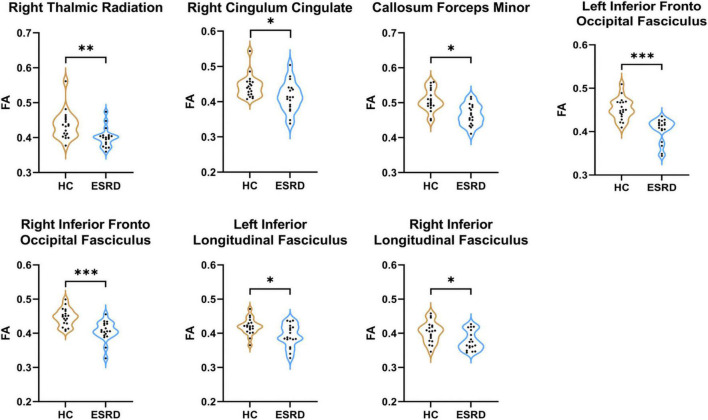
Fibers with statistical differences in mean FA values between HCs and ESRD patients. *P < 0.05, ^**^P < 0.01, ^***^P < 0.001.

For the whole global tract, compared to HCs, lower mean FA values were in the ESRD patients in the right ATR, right cingulum cingulate (CC), bilateral IFOF, bilateral ILF, and callosum forceps minor (*P* < 0.05, FDR correction, Supplementary Table 1 and Figure 2).

**FIGURE 3:**
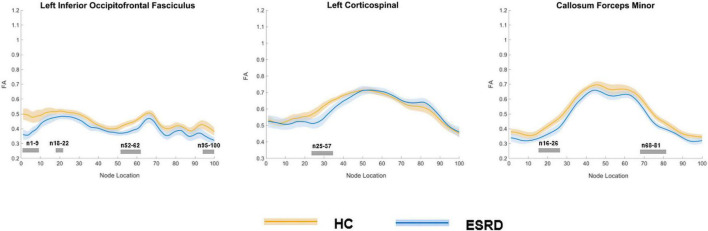
Plots of significantly altered locations in point-wise comparison of FA values between HCs and ESRD (*P* < 0.05, FDR correction). The orange line represents the HC group; the blue line represents the ESRD group (solid lines for means and shaded regions for confidence interval). The gray bars at the bottom are the regions of the fiber segment with significant difference between the two groups. ESRD, end stage renal disease; HC, health control; FA, fractional anisotropy.

In the point-wise comparison between groups, the positions of the fiber bundles that changed significantly were as follows: (a) the anterior and posterior component of the left IFOF (nodes 1–9; nodes 18–22; nodes 52–62; and nodes 95–100); (b) the inferior left corticospinal (CST; nodes 25–37); and (c) the frontal and occipital portion of the callosum forceps minor (nodes 16–26 and nodes 68–81; Figure 3, *P* < 0.05, FDR correction).

#### Mean Diffusivity

**FIGURE 4:**
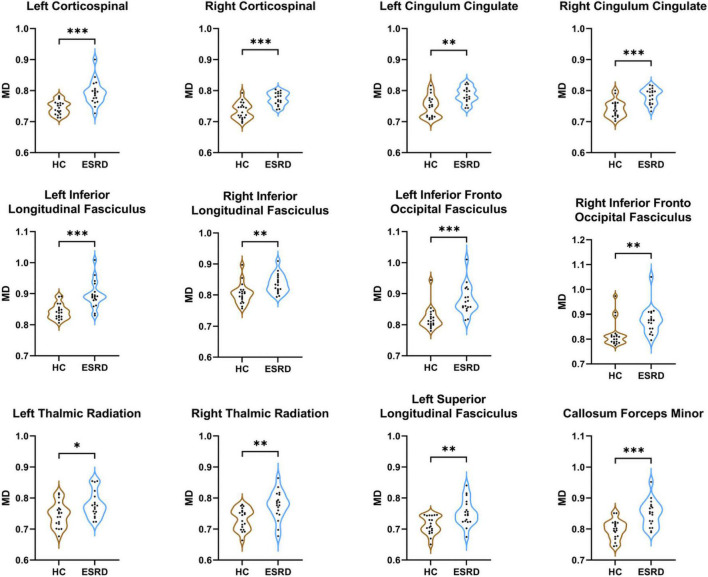
Fibers with statistical differences in mean MD values between HCs and ESRD patients. *P < 0.05, **P < 0.01, ***P < 0.001.

Compared to HC, ESRD group showed significantly increased MD value in multiple fiber bundles, including the bilateral CST, CC, ILF, IFOF, and ATR, the left SLF and callosum forceps minor (*P* < 0.05, FDR correction, Supplementary Table 2 and Figure 4).

**FIGURE 5:**
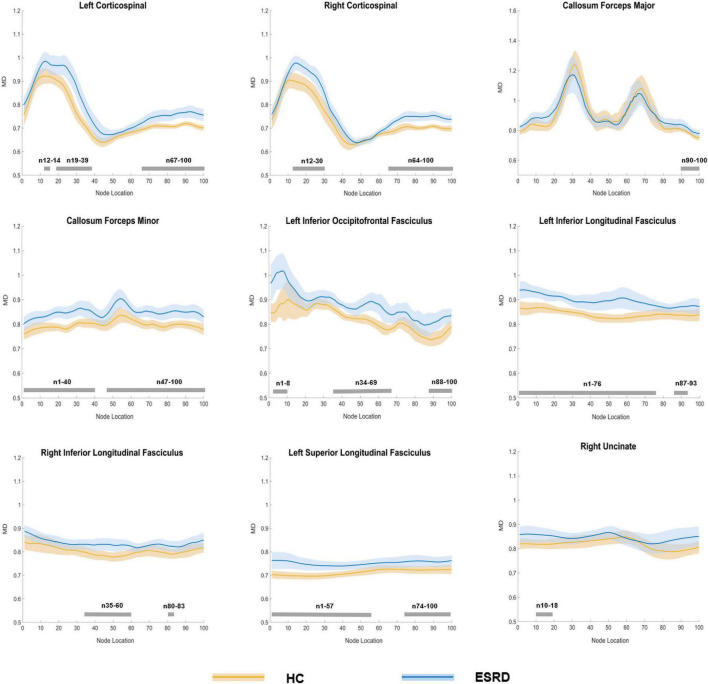
Plots of significantly altered locations in point-wise comparison of MD values between HCs and ESRD (*P* < 0.05, FDR correction). The orange line represents the HC group; the blue line represents the ESRD group (solid lines for means and shaded regions for confidence interval). The gray bars at the bottom are the regions of the fiber segment with significant difference between the two groups. ESRD, end stage renal disease; HC, health control; MD, mean diffusivity.

In the point-wise comparison of MD values, the significantly altered fiber bundle locations (*P* < 0.05, FDR correction) were as follows: (a) the inferior and superior left CST (nodes 12–14; nodes 19–39; and nodes 67–100) and right CST (nodes 12–30 and nodes 64–100); (b) the occipital portion of the forceps major (nodes 90–100) and the widespread distribution of the callosum forceps minor (nodes 1–40 and nodes 47–100); (c) the anterior, intermediate, and posterior parts of the left IFOF (nodes 1–8; nodes 34–69; and nodes 88–100); (d) wide distribution of the biliteral ILF (L: nodes 1–76 and nodes 87–93; R: nodes 35–60 and nodes 80–83); (e) the widespread distribution of the left SLF (nodes 1–57 and nodes 74–100); and (f) the occipital lobe component of the right uncinate fasciculus (UF, nodes 10–18; Figure 5).

#### Axial Diffusivity

In comparison with the HCs, the ESRD group exhibited significantly greater AD values in the bilateral CST, left CC, and bilateral ILF (FDR correction, Supplementary Table 3 and Supplementary Figure 1).

Point-wise comparison showed that the specific location changes of fibers (*P* < 0.05, FDR correction) as follows: (a) the superior parts of the left CST (nodes 66–100) and right CST (nodes 67–96) and (b) the posterior component of the left ILF (nodes 64–66; Supplementary Figure 2).

#### Radial Diffusivity

In the fiber tract level, the callosum forceps minor, bilateral CST, bilateral CC, bilateral IFOF, bilateral ILF, left SLF, right ATR, and right UF exhibited significant differences between HC and ESRD groups (FDR correction, Supplementary Table 4 and Supplementary Figure 3).

The following fibers had significant changes in the point-wise comparison (*P* < 0.05, FDR correction), including (a) the inferior portion of the left CST (nodes 20–40) and right CST (nodes 12–29); (b) the frontal and occipital portion of the forceps major (nodes 14–18 and nodes 80–85) and the entire fiber bundle of the callosum forceps minor (nodes 1–100); (c) the widespread distribution of the left IFOF (nodes 1–9, nodes 19–22, nodes 32–44, nodes 49–67, and nodes 87–100); (d) the wide distribution of the left ILF (nodes 1–13, nodes 19–61, and nodes 89–95) and intermediate and posterior parts of the right ILF (nodes 34–60 and nodes 81–83); (e) the anterior part of the left SLF (nodes 4–27); and (f) the frontal component part of the right UF (nodes 94–100; Supplementary Figure 4).

### Correlation Analysis

#### Correlations Between Diffusion Metrics and Montreal Cognitive Assessment and Biochemical Tests

Among the 12 ESRD patients who completed MoCA assessment, partial correlation analysis between MoCA and diffusion metrics of each fiber cluster where group differences emerged or mean values of fiber tracts were performed.

We found highly positive correlation between visuospatial/executive score and the mean FA values of the right ATR (*r* = 0.823, *P* = 0.012); the mean AD values of the left CC were negatively related to total MoCA score (*r* = −0.836, *P* = 0.010) and visuospatial/executive score (*r* = -0.898, *P* = 0.002). Significant negative correlations were discovered between the RD values of the occipital component part of the callosum forceps major and naming score (nodes 14–18, *r* = −0.748, *P* = 0.033; Supplementary Figure 5).

See Supplementary Figure 6 for the details of the partial correlation analysis between biochemical tests and diffusion metrics.

#### Multiple Linear Regression Analysis

The multiple regression analysis revealed that urea and UA level were independent predictors of the AD value of superior component of the left CST. Besides, we also found that urea level was the independent predictors of mean MD value of left ATR (Table 2).

**TABLE 2:** Multiple linear regression analysis.

	β	*P*
**AD value of N66-100 of left CST**		
Urea	0.407	0.007
UA	0.311	0.000
**Mean MD value of left ATR**		
Urea	0.528	0.034

*ATR, anterior thalamic radiation; AD, axial diffusivity; MD, mean diffusivity; CST, corticospinal tract; Urea, serum urea; Cre, creatinine; UA, uric acid.*

## Discussion

In this study, we first investigate the specific alterations of white matter microstructure in patients with ESRD by AFQ and further explored the relationships of white matter integrity with MoCA scores and biochemical indicators. We found the following points: (1) ESRD patients had extensive microstructure fragility of white matter fiber bundles, especially on the left side of the hemisphere; (2) changes of white matter microstructure might not only occur along the entire white matter fiber bundle, but also at some specific fiber segments; and (3) the point-wise level analysis was more sensitive than fiber tract level analysis and white matter fiber damage might be associated with clinical symptoms and cognitive function.

There are several mechanisms for the development of cognitive dysfunction related to white matter microstructure fragility in ESRD patients. First, the accumulation of neurotoxins caused by dialysis or uremia can cause central nervous system complications (Smogorzewski, 2001). Second, asymptomatic cerebral infarction, white matter lesions, and microhemorrhage caused by degenerative microvascular disease can cause vascular dementia (Arismendi-Morillo and Fernández-Abreu, 2010). Third, hemodialysis leads to changes in cerebral blood flow, leading to brain tissue and metabolic disorders (Kanai et al., 2001). Fourth, increased oxidative stress and chronic inflammation in ESRD may lead to endothelial and neuronal damage (Bugnicourt et al., 2013).

In our research, we applied four diffusion metrics to evaluate the alterations in the microstructure of white matter fiber bundles. FA and MD reflect the overall directionality and magnitude of water diffusion, respectively (Le Bihan et al., 2001). RD and AD denote the extent of diffusion perpendicular and parallel to fiber orientations, respectively (Assaf and Pasternak, 2008). All these metrics are purported to be sensitive to myelin and axon damage. Therefore, by using MD, AD, and RD values together with the FA value, white matter changes in ESRD patients can be better characterized. In comparison with HCs at the overall fiber level, ESRD patients exhibited widespread damaged tract profiles, with significant FA reduction in 43.75% (7/16) and MD, AD, and RD increase in 75% (12/16), 31.25% (5/16), and 75% (12/16) of the fiber tracts examined, respectively. And point-wise level analysis allowed us to obtain more accurate damaged segments, especially in MD and RD. These damage segments were mainly observed on fibers included projection fiber (CST), commissural fibers (forceps minor, forceps major), and association fibers (IFOF, ILF, SLF), especially for the association fibers, which have changed in all the four diffusion metrics. IFOF, as the longest associative bundle, is an important link connecting the frontal, temporal, and occipital lobes (Caverzasi et al., 2014) and plays a major role in the neuromotor function and processing of vison and hearing. Studies have found that the loss of WM integrity in IFOF have been considered a risk factor for Alzheimer’s disease, and Alzheimer’s disease patients show extensive damage along the IFOF bundle, including the anterior, posterior, and central region associated with memory impairment (Fu et al., 2014; Chen H. F. et al., 2020). Our findings indicated that ESRD patients have extensive damage to the left IFOF, including the anterior, posterior, and central regions, and mainly characterized in the form of decreased FA values and increased MD values, indicating that the microstructure changes of IFOF are mainly myelin damage. ILF is a critical pathway that primarily connects the occipital cortex and temporal lobe. Impaired integrity of ILF and SLF is associated with language comprehension and attention-deficit disorders (Wolfers et al., 2015; Shin et al., 2019). These results are similar to previous TBSS studies on CKD (Yin et al., 2018; Liu et al., 2020), while more detailed localization information of damaged segments of fibers can be determined in our current study based on AFQ analyses. Meanwhile, these results indicated that MD and RD values are more sensitive than FA in discovering the extent of white matter microstructure vulnerability, which consistent with previous studies (Amlien and Fjell, 2014; Jin et al., 2017; Chen H. F. et al., 2020). In addition, this study found that almost all segments of forceps minor had abnormal white matter microstructures, and with the accumulation of urea, the microstructure of nodes 16–26 in the forceps minor tended to damage. The corpus callosum is composed of white matter tracts that connect the left and right cerebral hemispheres and is the main commissural region of the brain. And the forceps minor is the fiber that connects the lateral and medial regions of the bilateral frontal lobes, which is involved in processing speed (Kerchner et al., 2012). Patients with ESRD can experience cognitive changes in different aspects, and the decline in executive function is the most obvious (Berger et al., 2016). Based on the point of view, this study found that with the decline of renal function, the microstructure of the frontal part of the forceps minor may be damaged, which may lead to impaired cognitive function. Unfortunately, our study did not carry out tests related to executive function, and further research is needed.

Besides, our study found that most of the damaged fiber bundles were in the left hemisphere, suggesting that the white matter vulnerability may have hemispheric heterogeneity. Previous study on microstructure damage in Alzheimer’s disease has also found heterogeneity in brain structure changes between hemispheres (Chen H. et al., 2020). At present, studies have shown that patients with CKD are more prone to result in cognitive decline and Alzheimer’s disease (Etgen et al., 2012; Zhang et al., 2020). Therefore, we speculate that these changes in brain white matter structure of ESRD patients may be related to the occurrence and development of Alzheimer’s disease.

In addition, this study found that changes in the diffusion metrics of ATR were related to the decline in cognitive level, especially visuospatial/executive. The ATR, which connects the mediodorsal and anterior thalamic nuclei (ATN) with the frontal cortex and the anterior cingulate cortex, can process incoming information from the hippocampus (related to cognitive functions) (Setiadi et al., 2021). Therefore, the decreased integrity of ATR may be associated with decreased cognitive functions. Different from the hemispheric heterogeneity reflected by the comparison between groups, we found that the cognitive-related ATR white matter damage were mainly located in the right hemisphere, which may be related to the priority of damage first appearing in the dominant hemisphere. Besides, a study related to reduced attention of young bilingual adults showed that the integrity of the white matter fibers connecting the anterior cingulate cortex and the frontal regions was decreased, and this manifestation only exists in the right hemisphere (Mamiya et al., 2018). Similarly, Huang et al. (2020) also reported that the right ATR was associated with executive function in cerebral small vessel disease patients.

Excitingly, this work found that specific white matter segments were closely associated with specific laboratory test results, such as urea, UA, and Cre. In particular, through multiple linear regression analysis, these specific segments corresponding to specific renal function indexes were determined. Put simply, renal dysfunction in ESRD patients may mediate the corresponding specific white matter segment abnormalities. In this study, we found that with the increase of urea level, mean AD value of the left ATR and nodes of 66–100 in the left CST tended to be damaged. Besides, our study showed positive correlations of urea level with the increased MD in the left ATR. Our interpretation of this result is that impaired renal function can result in the continuous accumulation of metabolites, which in turn leads to demyelination and axonal damage (Mogi and Horiuchi, 2011; Lu et al., 2015). Previous TBSS studies on ESRD have also demonstrated that serum creatinine is related to white matter integrity (Kim et al., 2011; Yin et al., 2018).

Despite the advantages of this method, we should not ignore some limitations of this study. First, our prospective study was conducted on a relatively small sample, and further investigations on relatively large population are still needed. Second, due to the threshold setting in fiber tracking, some fibers such as the CH and AF were adjacent to the gray matter, which makes them impossible to be tracked. Third, in this study, we only focused on patients with ESRD, and in the future, patients with early CKD should be enrolled to explore the correlation between disease progression and white matter microstructure changes. Fourth, due to participant compliance, not all participants had completed the cognitive assessments, which may have a certain impact on the relevant results.

## Conclusion

In conclusion, this study identified specific abnormalities in major white matter fibers in ESRD using a tract profile approach. Besides, the results of our study indicates that the accumulation of serum urea and uric acid may lead to vulnerable white matter microstructure. We believe that the findings of this work will enhance our understanding of white matter abnormalities in ESRD.

## Data Availability Statement

The raw data supporting the conclusions of this article will be made available by the authors, without undue reservation.

## Ethics Statement

This study was approved by the Ethics Committee of the First Affiliated Hospital of Dalian Medical University. The patients/participants provided their written informed consent to participate in this study. Written informed consent was obtained from the individual(s) for the publication of any potentially identifiable images or data included in this article.

## Author Contributions

YJ and YL: guarantor of integrity of entire study. YJ, YL, BG, YC, JJ, and PC: literature research. YJ, YL, BG, YC, JJ, PC, QS, LL, NW, WW, and YM: clinical studies. YJ, YL and YM: experimental studies. YJ, YL, and JJ: statistical analysis. YJ, YL, LL, and YM: manuscript editing. All authors: study concepts, study design, data acquisition, data analysis, interpretation, manuscript drafting, manuscript revision for important intellectual content, approval of final version of submitted manuscript, agrees to ensure any questions related to the work are appropriately resolved.

## Conflict of Interest

LL was employed by the company Philips Healthcare China. The remaining authors declare that the research was conducted in the absence of any commercial or financial relationships that could be construed as a potential conflict of interest.

## Publisher’s Note

All claims expressed in this article are solely those of the authors and do not necessarily represent those of their affiliated organizations, or those of the publisher, the editors and the reviewers. Any product that may be evaluated in this article, or claim that may be made by its manufacturer, is not guaranteed or endorsed by the publisher.

## Footnotes

1
https://github.com/vistalab/vistasoft

2
http://fsl.fmrib.ox.ac.uk/fsl/

## Abbreviations

AFQ: automated fiber quantification
AD: axial diffusivity
ATR: anterior thalamic radiation
CST: corticospinal
Cre: Creatinine
ESRD: end stage renal disease
FA: fractional anisotropy
FDR: false discovery rate
IFOF: inferior fronto-occipital fasciculus
ILF: inferior longitudinal fasciculus
MD: mean diffusivity
MoCA: Montreal Cognitive Assessment
RD: radial diffusivity
SLF: superior longitudinal fasciculus
Urea: serum urea
UA: uric acid.
