# Supplementary material for: Segmental Abnormalities of White Matter Microstructure in End-Stage Renal Disease Patients: An Automated Fiber Quantification Tractography Study
Source: Front Neurosci. 2021 Dec 6;15:765677. doi: 10.3389/fnins.2021.765677 (PMC8685541; doi:10.3389/fnins.2021.765677)
Supplement: Supplementary file 1 [file Data_Sheet_1.DOCX]

**Supplementary Materials**

| **Supplementary Table S1 Mean FA values (× 100) for HC and ESRD groups** | | | | | | | | | | |  |  |
| --- | --- | --- | --- | --- | --- | --- | --- | --- | --- | --- | --- | --- |
|  |  |  |  |  |  |  |  |  |  |  |  |  |
| **Index** | **Tract** | | **Group** | | | | ***t*** | | ***p*** | |  |  |
|  |  |  | **HC** | | **ESRD** | |  |  |  |  |  |  |
| 1 | ATR_L | | 0.408 | | 0.400 | | 0.6310 | | 0.5570 | |  |  |
| 2 | ATR_R | | 0.436 | | 0.401 | | 3.1010 | | 0.010* | |  |  |
| 3 | CST_L | | 0.607 | | 0.598 | | 0.7740 | | 0.4820 | |  |  |
| 4 | CST_R | | 0.603 | | 0.597 | | 0.6100 | | 0.5630 | |  |  |
| 5 | CC_L | | 0.461 | | 0.446 | | 1.0750 | | 0.3430 | |  |  |
| 6 | CC_R | | 0.443 | | 0.414 | | 2.3870 | | 0.041* | |  |  |
| 7 | Forceps major | | 0.571 | | 0.554 | | 1.0570 | | 0.3430 | |  |  |
| 8 | Forceps minor | | 0.507 | | 0.465 | | 4.1370 | | 0.001* | |  |  |
| 9 | IFOF_L | | 0.452 | | 0.404 | | 5.5980 | | 0.0001* | |  |  |
| 10 | IFOF_R | | 0.447 | | 0.406 | | 4.4880 | | 0.0005* | |  |  |
| 11 | ILF_L | | 0.420 | | 0.392 | | 2.9150 | | 0.015* | |  |  |
| 12 | ILF_R | | 0.404 | | 0.379 | | 2.5820 | | 0.0286* | |  |  |
| 13 | SLF_L | | 0.430 | | 0.418 | | 0.7700 | | 0.4817 | |  |  |
| 14 | SLF_R | | 0.463 | | 0.447 | | 1.0700 | | 0.3432 | |  |  |
| 15 | UF_L | | 0.405 | | 0.388 | | 1.7000 | | 0.1310 | |  |  |
| 16 | UF_R | | 0.406 | | 0.389 | | 1.6270 | | 0.1473 | |  |  |
| Values are presented as the mean ± standard deviation (SD) | | | | | | | | | | |  |  |
| * Indicates a statistical difference between groups, *P* < 0.05 | | | | | | | | | | |  |  |
| Abbreviation: HC, health control; ESRD, end stage renal disease; FA, fractional anisotropy; ATR_L, left anterior thalamic radiation; ATR_R, right anterior thalamic radiation; CST_L, left corticospinal tract; CST_R, right corticospinal tract; CC_L, left cingulum cingulate; CC_R, right cingulum cingulate; IFOF_L, left inferior fronto-occipital fasciculus; IFOF_R, right inferior fronto-occipital fasciculus; ILF_L, left inferior longitudinal fasciculus; ILF_R, right inferior longitudinal fasciculus; SLF_L, left superior longitudinal fasciculus; SLF_R, right superior longitudinal fasciculus; UF_L, left uncinate fasciculus; UF_R, right uncinate fasciculus. | | | | | | | | | | |  |  |
| **Supplementary Table S2 Mean MD values ( × 100) for HC and ESRD groups** | | | | | | | | | | | |  |
|  |  |  |  |  |  |  |  |  |  |  |  |  |
| **Index** | | **Tract** | | **Group** | | | | ***t*** | | ***p*** | |  |
|  |  |  |  | **HC** | | **ESRD** | |  |  |  |  |  |
| 1 | | ATR_L | | 0.746 | | 0.779 | | -2.436 | | 0.0386* | |  |
| 2 | | ATR_R | | 0.732 | | 0.776 | | -3.319 | | 0.0063* | |  |
| 3 | | CST_L | | 0.745 | | 0.793 | | -4.725 | | 0.0004* | |  |
| 4 | | CST_R | | 0.735 | | 0.774 | | -5.018 | | 0.0003* | |  |
| 5 | | CC_L | | 0.748 | | 0.786 | | -3.835 | | 0.002* | |  |
| 6 | | CC_R | | 0.741 | | 0.783 | | -4.933 | | 0.0003* | |  |
| 7 | | Forceps major | | 0.908 | | 0.915 | | -0.327 | | 0.7566 | |  |
| 8 | | Forceps minor | | 0.798 | | 0.849 | | -4.285 | | 0.0007* | |  |
| 9 | | IFOF_L | | 0.821 | | 0.880 | | -4.367 | | 0.0006* | |  |
| 10 | | IFOF_R | | 0.819 | | 0.876 | | -3.222 | | 0.0076* | |  |
| 11 | | ILF_L | | 0.842 | | 0.896 | | -4.285 | | 0.0004* | |  |
| 12 | | ILF_R | | 0.804 | | 0.836 | | -3.092 | | 0.0102* | |  |
| 13 | | SLF_L | | 0.713 | | 0.753 | | -3.397 | | 0.0053* | |  |
| 14 | | SLF_R | | 0.705 | | 0.727 | | -2.019 | | 0.0791 | |  |
| 15 | | UF_L | | 0.826 | | 0.853 | | -2.093 | | 0.0690 | |  |
| 16 | | UF_R | | 0.819 | | 0.846 | | -2.222 | | 0.0558 | |  |
| Values are presented as the mean ± standard deviation (SD)  * Indicates a statistical difference between groups, *P* < 0.05 | | | | | | | | | | | |  |

Abbreviation: HC, health control; ESRD, end stage renal disease; MD, mean diffusivity; ATR_L, left anterior thalamic radiation; ATR_R, right anterior thalamic radiation; CST_L, left corticospinal tract; CST_R, right corticospinal tract; CC_L, left cingulum cingulate; CC_R, right cingulum cingulate; IFOF_L, left inferior fronto-occipital fasciculus; IFOF_R, right inferior fronto-occipital fasciculus; ILF_L, left inferior longitudinal fasciculus; ILF_R, right inferior longitudinal fasciculus; SLF_L, left superior longitudinal fasciculus; SLF_R, right superior longitudinal fasciculus; UF_L, left uncinate fasciculus; UF_R, right uncinate fasciculus.

| **Supplementary Table S3 Mean AD values ( × 100) for HC and ESRD groups** | | | | | |
| --- | --- | --- | --- | --- | --- |
|  |  |  |  |  |  |
| **Index** | **Tract** | **Group** | | ***t*** | ***p*** |
|  |  | **HC** | **ESRD** |  |  |
| 1 | ATR_L | 1.100 | 1.142 | -2.197 | 0.0576 |
| 2 | ATR_R | 1.110 | 1.139 | -1.482 | 0.1853 |
| 3 | CST_L | 1.336 | 1.409 | -3.916 | 0.0021* |
| 4 | CST_R | 1.311 | 1.373 | -4.711 | 0.0004* |
| 5 | CC_L | 1.168 | 1.210 | -2.328 | 0.045* |
| 6 | CC_R | 1.137 | 1.162 | -1.723 | 0.1304 |
| 7 | Forceps major | 1.589 | 1.580 | 0.296 | 0.7692 |
| 8 | Forceps minor | 1.315 | 1.344 | -1.738 | 0.1304 |
| 9 | IFOF_L | 1.268 | 1.298 | -1.711 | 0.1304 |
| 10 | IFOF_R | 1.253 | 1.287 | -1.536 | 0.1721 |
| 11 | ILF_L | 1.256 | 1.306 | -2.957 | 0.015* |
| 12 | ILF_R | 1.173 | 1.191 | -0.976 | 0.0286* |
| 13 | SLF_L | 1.066 | 1.111 | -2.412 | 0.4817 |
| 14 | SLF_R | 1.088 | 1.110 | -1.176 | 0.3432 |
| 15 | UF_L | 1.220 | 1.239 | -1.25 | 0.2715 |
| 16 | UF_R | 1.212 | 1.229 | -0.974 | 0.3753 |
| Values are presented as the mean ± standard deviation (SD) | | | | | |
| * indicates a statistical difference between groups, *P* < 0.05 | | | | | |

Abbreviation: HC, health control; ESRD, end stage renal disease; AD, axial diffusivity; ATR_L, left anterior thalamic radiation; ATR_R, right anterior thalamic radiation; CST_L, left corticospinal tract; CST_R, right corticospinal tract; CC_L, left cingulum cingulate; CC_R, right cingulum cingulate; IFOF_L, left inferior fronto-occipital fasciculus; IFOF_R, right inferior fronto-occipital fasciculus; ILF_L, left inferior longitudinal fasciculus; ILF_R, right inferior longitudinal fasciculus; SLF_L, left superior longitudinal fasciculus; SLF_R, right superior longitudinal fasciculus; UF_L, left uncinate fasciculus; UF_R, right uncinate fasciculus.


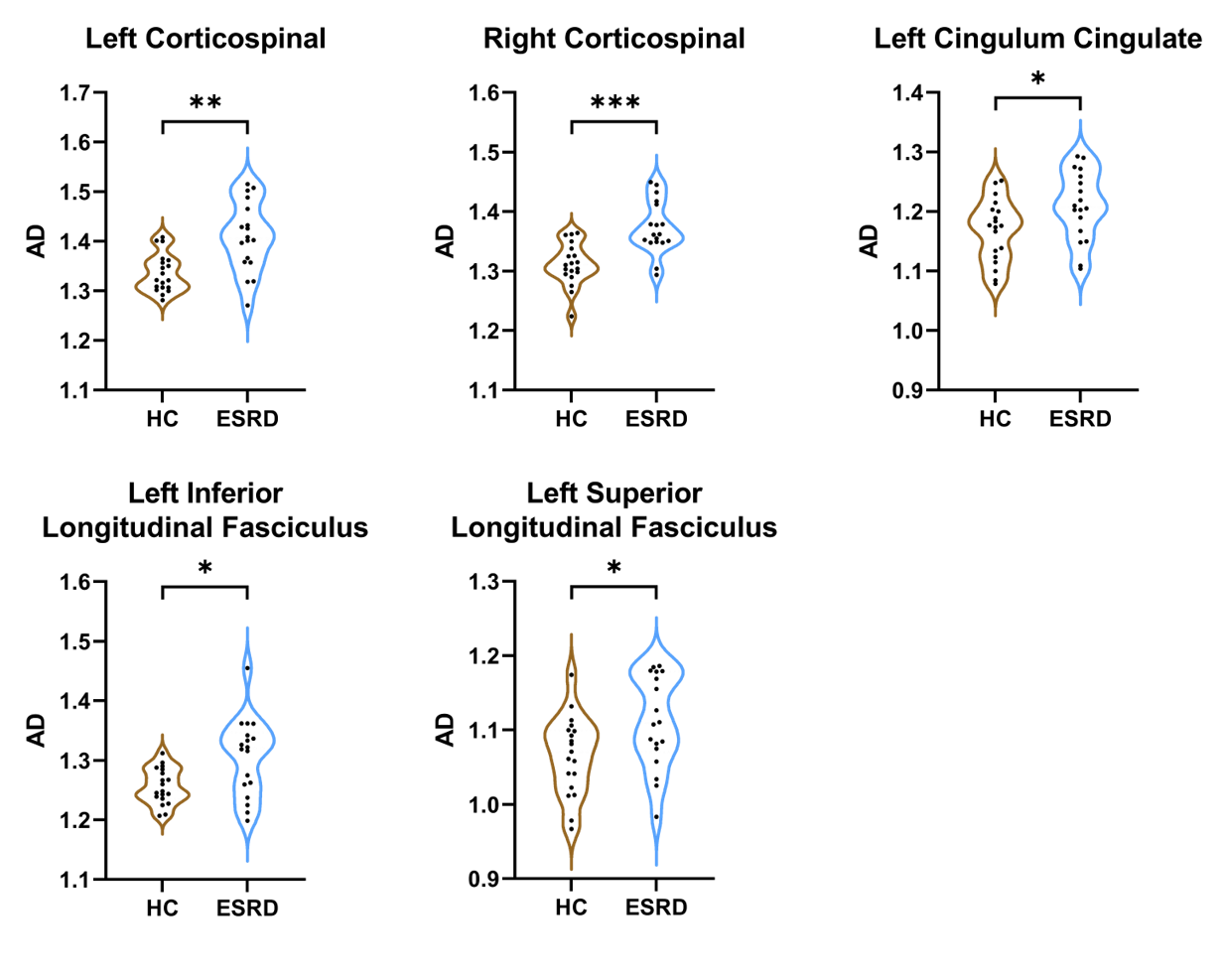


**Figure S1** Fibers with significant differences in mean AD values between the HC and ESRD groups. ∗*P*-value < 0.05, ∗∗*P*-value < 0.01, ∗∗∗*P*-value < 0.001.


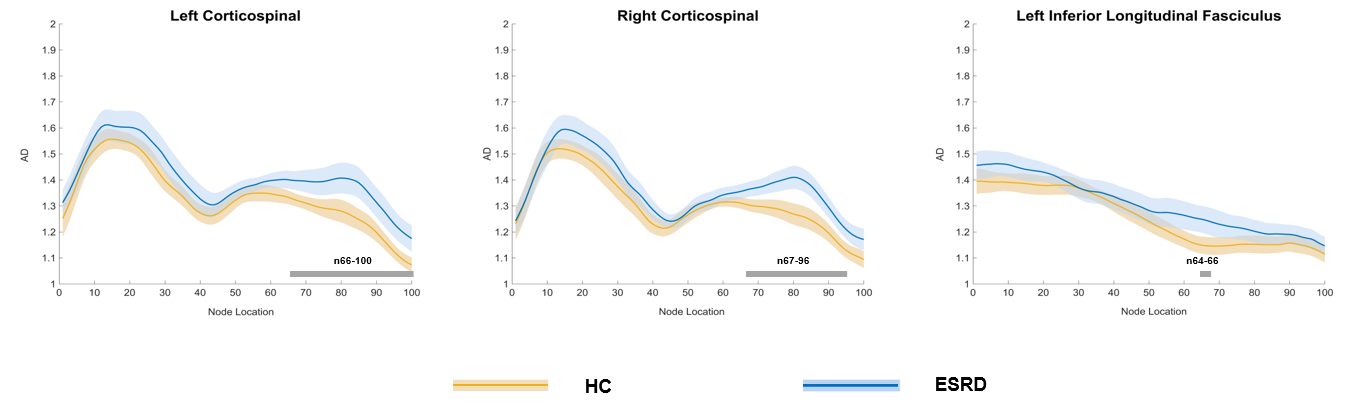


**Figure S2** Plots of significantly altered locations in point-wise comparison of AD profiles between HCs and ESRD (FDR correction, *P* < 0.05). The orange line represents the HC group, the blue line represents the ESRD group (solid lines for means and shaded regions for confidence interval). The grey bars at the bottom are the regions of the fiber segment with significant difference between the two groups. Abbreviation: HC, health control; ESRD, end stage renal disease; AD, axial diffusivity.

| **Supplementary Table 4 Mean RD values ( × 100) for HC and ESRD groups** | | | | | |
| --- | --- | --- | --- | --- | --- |
|  |  |  |  |  |  |
| **Index** | **Tract** | **Group** | | ***t*** | ***p*** |
|  |  | **HC** | **ESRD** |  |  |
| 1 | ATR_L | 0.569 | 0.598 | -1.891 | 0.0989 |
| 2 | ATR_R | 0.543 | 0.595 | -3.833 | 0.0020* |
| 3 | CST_L | 0.449 | 0.486 | -3.048 | 0.011* |
| 4 | CST_R | 0.446 | 0.474 | -2.753 | 0.0204* |
| 5 | CC_L | 0.538 | 0.574 | -2.88 | 0.0157* |
| 6 | CC_R | 0.543 | 0.593 | -4.415 | 0.0006* |
| 7 | Forceps major | 0.567 | 0.582 | -0.659 | 0.5461 |
| 8 | Forceps minor | 0.539 | 0.602 | -4.66 | 0.0004* |
| 9 | IFOF_L | 0.597 | 0.672 | -5.418 | 0.0002* |
| 10 | IFOF_R | 0.602 | 0.670 | -3.99 | 0.0015* |
| 11 | ILF_L | 0.635 | 0.691 | -4.41 | 0.0006* |
| 12 | ILF_R | 0.619 | 0.659 | -3.675 | 0.0027* |
| 13 | SLF_L | 0.536 | 0.573 | -2.645 | 0.0251* |
| 14 | SLF_R | 0.514 | 0.536 | -1.72 | 0.1304 |
| 15 | UF_L | 0.629 | 0.660 | -2.099 | 0.0690 |
| 16 | UF_R | 0.623 | 0.655 | -2.338 | 0.045* |
| Values are presented as the mean ± standard deviation (SD) | | | | | |
| * indicates a statistical difference between groups, *P* < 0.05 | | | | | |

Abbreviation: HC, health control; ESRD, end stage renal disease; RD, radial diffusivity; ATR_L, left anterior thalamic radiation; ATR_R, right anterior thalamic radiation; CST_L, left corticospinal tract; CST_R, right corticospinal tract; CC_L, left cingulum cingulate; CC_R, right cingulum cingulate; IFOF_L, left inferior fronto-occipital fasciculus; IFOF_R, right inferior fronto-occipital fasciculus; ILF_L, left inferior longitudinal fasciculus; ILF_R, right inferior longitudinal fasciculus; SLF_L, left superior longitudinal fasciculus; SLF_R, right superior longitudinal fasciculus; UF_L, left uncinate fasciculus; UF_R, right uncinate fasciculus.


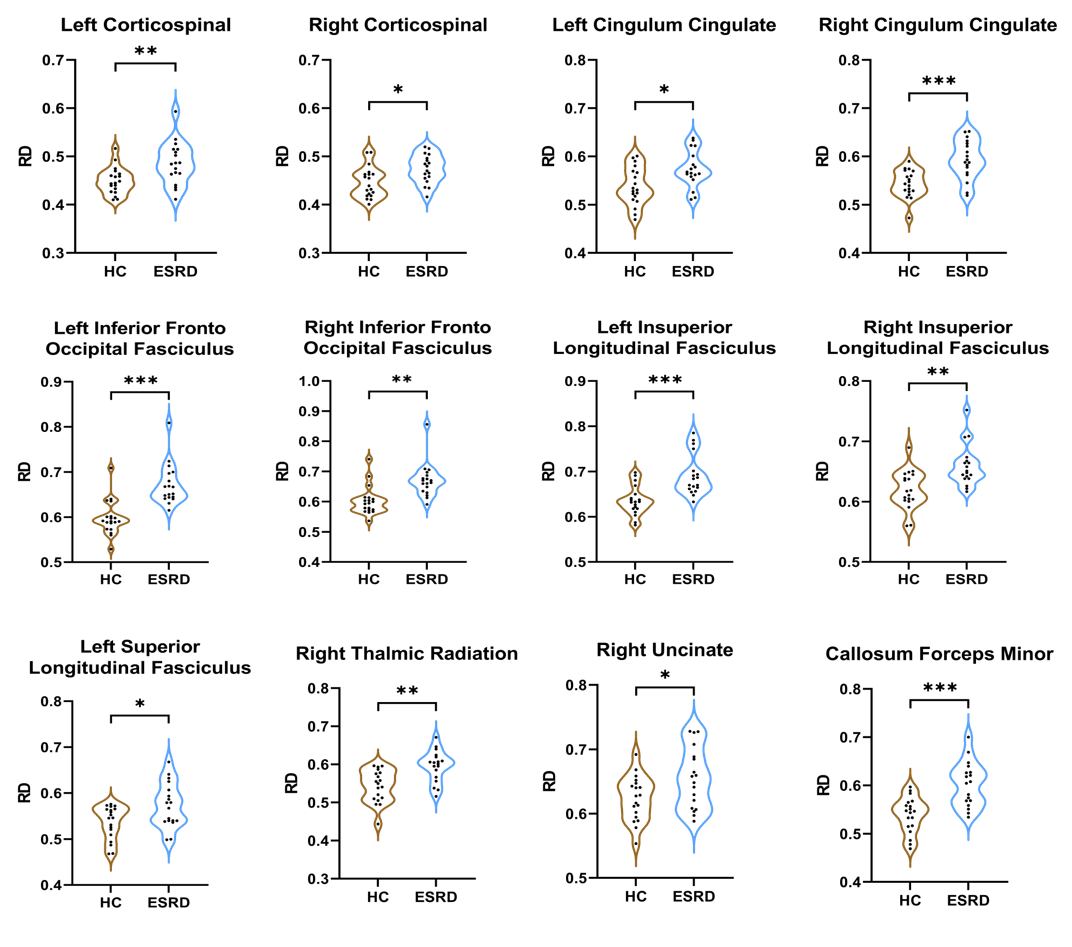


**Figure S3** Fibers with significant differences in mean RD values between the HC and ESRD groups ∗*P*-value < 0.05, ∗∗*P*-value < 0.01, ∗∗∗*P*-value < 0.001.


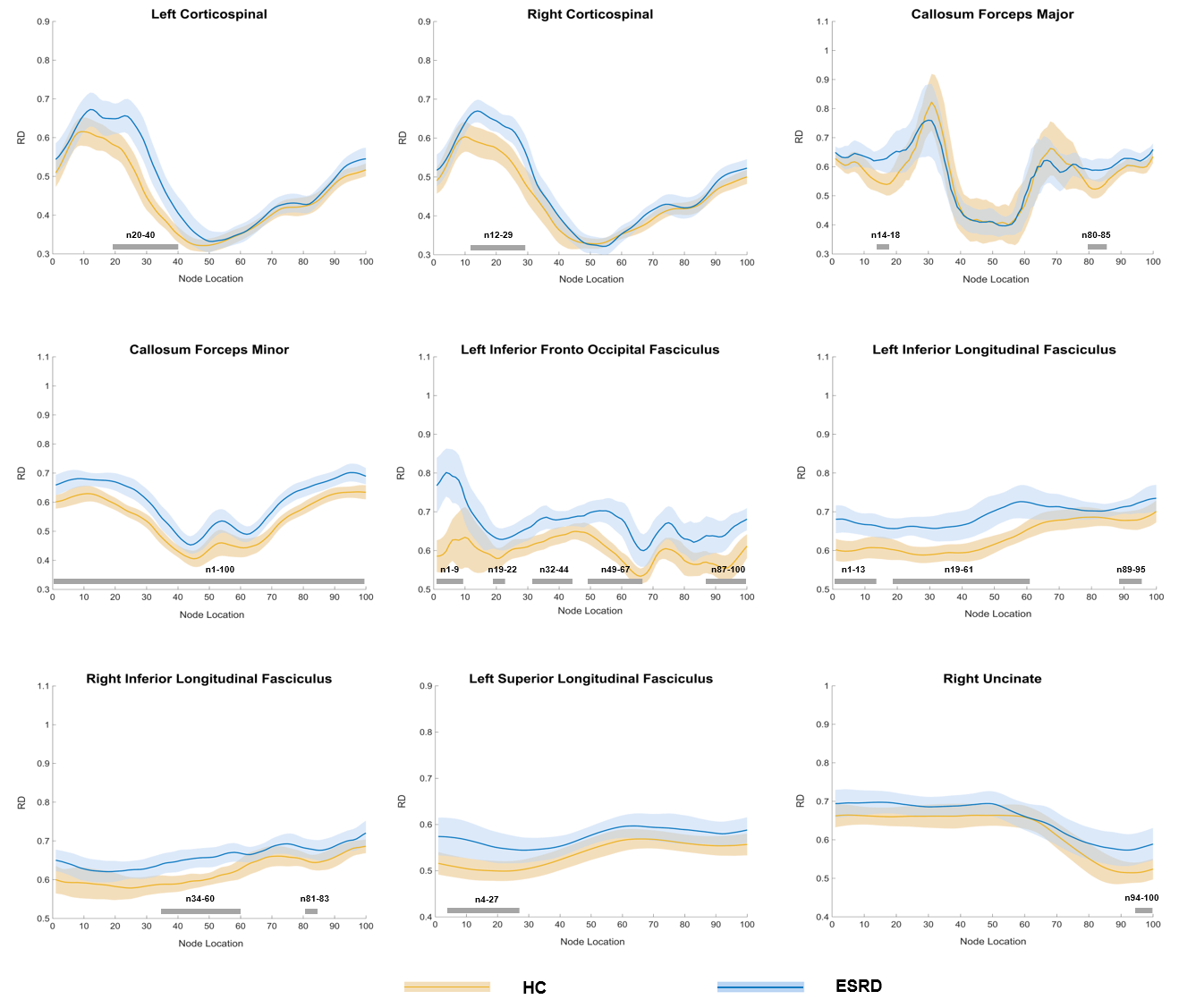


**Figure S4** Plots of significantly altered locations in point-wise comparison of RD profiles between HCs and ESRD (FDR correction, *P* < 0.05). The orange line represents the HC group, the blue line represents the ESRD group (solid lines for means and shaded regions for confidence interval). The grey bars at the bottom are the regions of the fiber segment with significant difference between the two groups. Abbreviation: HC, health control; ESRD, end stage renal disease; RD, radial diffusivity.

**
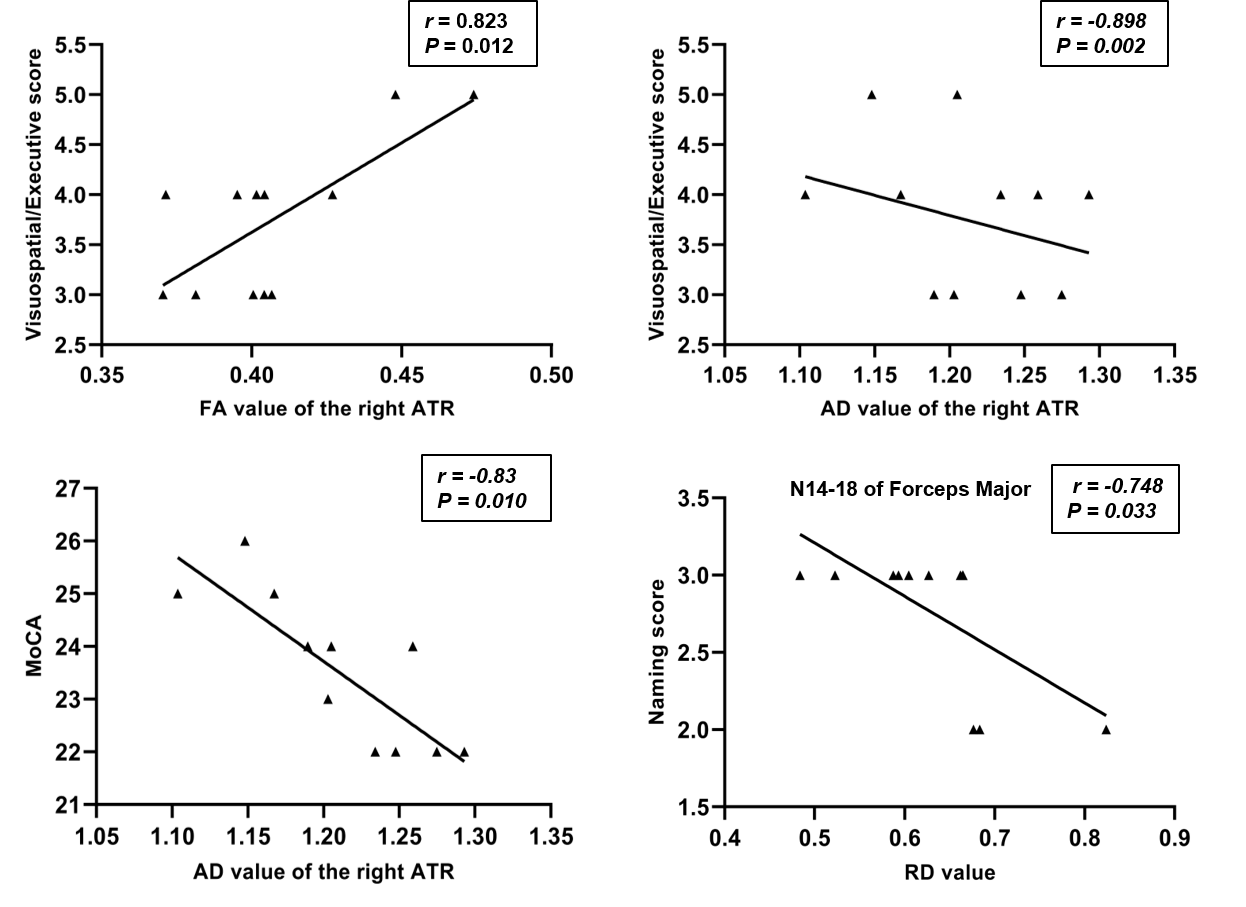
**

**Figure S5** Correlations between diffusion metrics and cognition assessments in ESRDs. ATR, anterior thalamic radiation; MoCA, Montreal Cognitive Assessment; FA, fractional anisotropy; AD, axial diffusivity; RD, radial diffusivity.

**3.3.1 Correlations between diffusion metrics and MoCA and biochemical tests**

In the whole ESRD group partial correlation analysis between biochemical tests and diffusion metrics, we found that Urea level was negatively related to the average FA value of the callosum forceps minor (nodes 16-26, *r* = -0.570, *P* = 0.033). Significant positive correlations between Urea and the mean MD values of the left CST (nodes 67-100, *r* = 0.617, *P* = 0.020) and left IFOF (nodes 88-100, *r* = 0.690, *P* = 0.006) were also observed. Besides, Urea had a positive relationship with mean AD values of the left CST (66-100, *r* = 0.569, *P* = 0.033), mean RD values of the left SLF (nodes 4-27, *r* = 0.654, *P* = 0.011) and right UF (nodes 94-100, *r* = 0.723, *P* = 0.004). Positive relationships were also observed between Cre and mean MD values of the left CST (nodes 19-39, *r* = 0.553, *P* = 0.040) and right CST (nodes 12-30, *r* = 0.559, *P* = 0.038). Meanwhile, we found significant positive correlation between UA and AD values of the left CST (nodes 66-100, *r* = 0.594, *P* = 0.025). (**Supplementary Figure S6**)

The correlations between biochemical tests and mean diffusion metrics of fiber tracts were also summarized. We noted positive correlations with mean MD in the following tracts: (a) Urea: left ATR (*r* = 0.653, *P* = 0.011), callosum forceps minor (*r* = 0.557, *P* = 0.039), right IFOF (*r* = 0.583, *P* = 0.029), right CST (*r* = 0.615, *P* = 0.019), left CST (*r* = 0.575, *P* = 0.032), left SLF (*r* = 0.589, *P* = 0.027); (b) Cre: left ATR (*r* = 0.636, *P* = 0.015), right IFOF (*r* = 0.615, *P* = 0.019), right CST (*r* = 0.676, *P* = 0.008), left CST (*r* = 0.58, *P* = 0.03). And mean AD values of the right CST and left CST had a positive correlation with Cre and UA, respectively (*r* = 0.566, *P* = 0.035; *r* = 0.572, *P* = 0.033). Mean RD of the bilateral IFOF had positive relationship with Urea (L: *r* = 0.560, *P* = 0.037; R: *r* = 0.574, *P* = 0.032). Unfortunately, the correlation results abovementioned did not survive the FDR correction (**Supplementary Figure S6**).


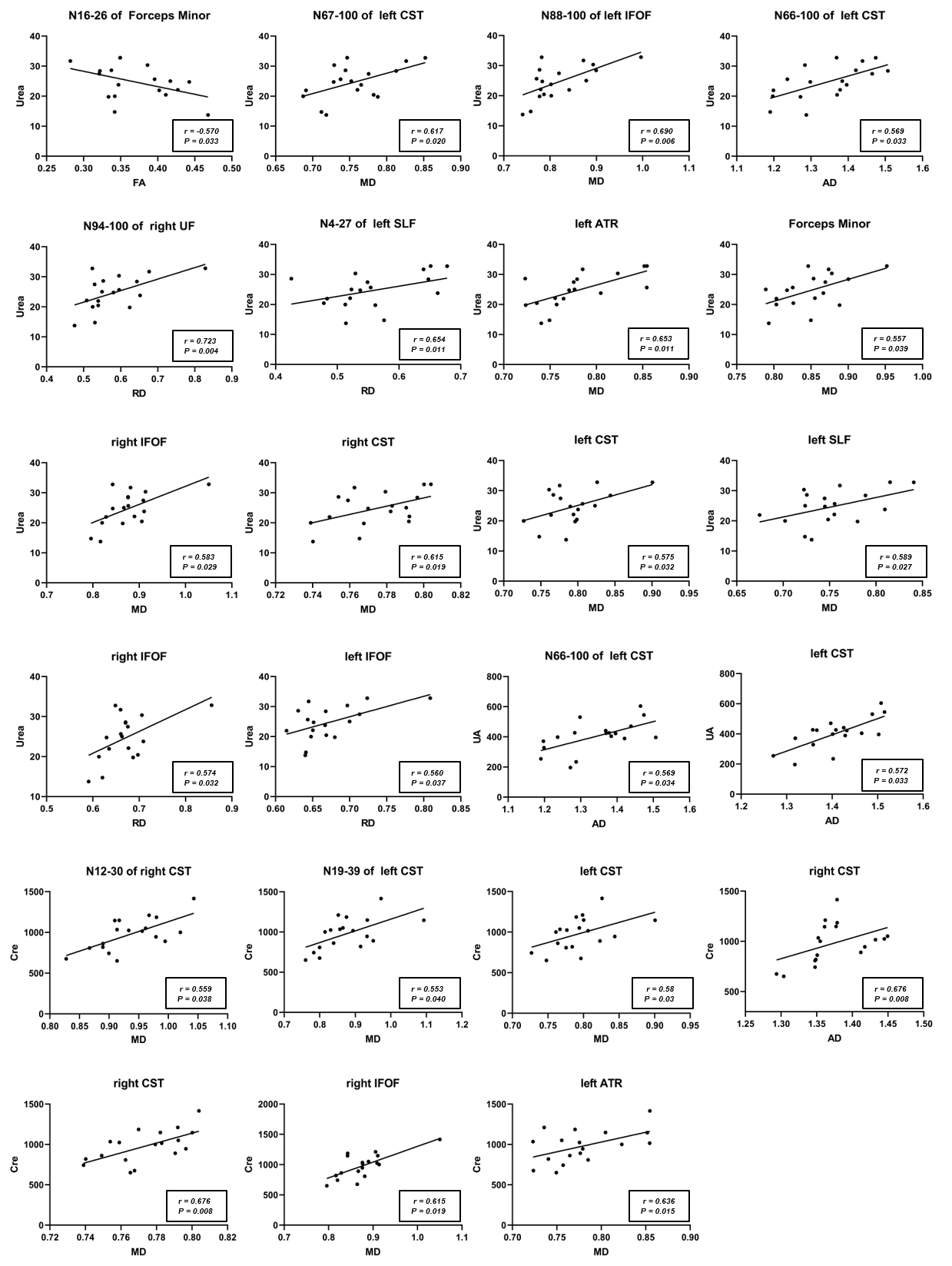


**Figure S6** Correlations between diffusion metrics and laboratory tests in ESRDs. SLF, superior longitudinal; ATR, anterior thalamic radiation; IFOF, inferior fronto‐occipital fasciculus; CST, corticospinal tract; UF, uncinate fasciculus; Urea, serum urea; Cre, creatinine; UA, uric acid.
